# Hydropathy; or the Cold Water Cure, as Practised by Vincent Priessnitz, at Gräeffenberg, in Silesia

**Published:** 1842-04-01

**Authors:** 


I. HYDROPATHY; OR THE COLD WATER CURE, AS PRACTISED BY VINCENT PRIESSNITZ, AT GRAEFFENBERG, IN SILESIA. By *R. T. Claridge*, Esq. Octavo, pp. 320. Madden & Co. London, 1842.

II. ETABLISSEMENT HYDRO-THERAPEUTIQUE DE MARIENBERG, PRES BOPPART SUR LE RHIN. Dr. *Schmitz*, 1842.

MR. CLARIDGE is mistaken in supposing that he was the first person to make known the "WATER CURE" of Priessnitz in this country. Dr. Johnson, more than a year ago, gave the pith of the matter, in the first edition of his "PILGRIMAGES TO THE SPAS," besides some short notices in this Journal. We should not be surprised to find hydropathy thrust aside homœopathy—"animal magnetism"—"brandy and salt"—or even "Morrison's pills." We understand that one, if not two establishments, are being formed in this country, for the cure of all diseases by perspiration and cold water—and that an eminent physician—distinguished for undoubted talent, unlimited belief, and boundless enthusiasm—a physician, who is always ready to adopt a new, and discard an old remedy—and who, like Paganini, has seldom more than one string to his fiddle—has become a disciple of Priessnitz, and will probably be the Apollo of Hydropathy in this country.

Dr. Schmitz, too, a respectable German physician, has set up a splendid hydropathic establishment, near Boppart on the Rhine, in the ancient monastery of *Marienberg*, with every advantage of air, water, and beautiful scenery. We should not be surprised if Marienberg attracts a considerable number of the Rhine-goers this Summer, were it only from curiosity to see this wonderful process for bleaching both the outside and inside of hypochondriacs and malades imaginaires.

Mr. Claridge must have an organ of credulity, beyond all measurement, if he really believes that the Silesian peasant, (who, according to Mr. C.'s own confession, does not know in which side of the abdomen the liver lies,) is "one of the greatest benefactors of mankind—one of the most astounding geniuses of this or any other age—the founder of a system, by which he proves, *beyond the power of contradiction*, that all *curable* diseases, and many declared by the faculty beyond the power of their art, are to be cured by the sole agency of cold spring water, air, and exercise." Why Morrison *proves* that he can do even more than all this by a single box of pills—while "brandy and salt," upon the very same kind of testimony, puts to flight every ill that flesh is heir to. Mr. C. tell us that the aid of this "SECOND HIPPOCRATES" "has been sought by upwards of 7000 invalids." This is very probable. Far more than that number have *sought* the aid of Morrison's pills—and a pretty considerable number of these have found a final cure for all their maladies—in their graves.

Mr. C. does not inform us very distinctly what was the precise nature of the complaint which induced him to take a pilgrimage to Gräefenberg. We should suppose it was not a very serious one; nor do we imagine that many of the "two or three hundred" who daily sat down to dinner at the hydropathic table-d'hôte, were affected with very *grave* maladies. Our author "could not help remarking the happy, healthy-looking countenances of *all* around, and the merry laugh and mirth which burst from every part of the large saloon." Whoever has taken his stand, for a couple of hours, at the Cockbrunnen, Sprudel, or other efficient source of mineral waters, and observed the squalid invalids that encircle them, will be not a little surprised to learn from the author before us, that the jolly jovial crew assembled at Gräeffenberg, "come here after having consulted *all* the celebrated doctors within their reach, and tried the mineral waters of Germany in vain." Is it not astonishing that a writer, of even the most moderate share of discernment, should not be struck at once with the utter inconsistency—not to say the utter incompatibility, of the two statements adduced above!

The following is the formula through which Mr. Claridge himself passed, which may answer as a specimen of hydropathy.

"At four o'clock in the morning, my servant folded me in a large blanket, over which he placed as many things as I could conveniently bear; so that no external air could penetrate. After perspiration commenced, it was allowed to continue for an hour: he then brought a pair of straw shoes, wound the blanket close about my body, and in this state of perspiration I descended to a large cold-bath in which I remained three minutes; then dressed and walked until breakfast, which was composed of milk, bread, butter, and strawberries, (the wild strawberry in this country grows in abundance, from the latter end of May until late in October;) at ten o'clock I proceeded to the douche, under which I remained four minutes, returned home, and took a sitz and foot-bath, each for fifteen minutes; dined at one o'clock; at four proceeded again to the douche; at seven repeated the sitz and foot-baths; retired to bed at half-past nine, previously having my feet and legs bound up in cold wet bandages. I continued this treatment for three months, and, during that time, walked about 1000 miles. Whilst thus subjected to the treatment, I enjoyed more robust health than I had ever done before; the only visible effect that I experienced, was an eruption on both my legs, but which, on account of the bandages, produced no pain. It is to these bandages, the perspirations, and the baths, that I am indebted for the total departure of my rheumatism." 15.

Mr. Claridge is very much in error, when he thinks that this practice is original with Priessnitz. It will presently be seen that, as far as the bathing process is concerned, and it is the principal, the Russians have employed it time immemorial. The ingurgitation of large quantities of cold water is also an old practice, as everybody knows. The following condensed formula was published by Dr. J. Johnson more than a year ago.

"About four or five o'clock in the morning, the patient is wrapped up to the chin (while in bed) in a thick woollen shirt. Outside of this is placed another covering of down, fur, or any warm and impermeable material. In a short time the disengagement of animal heat from the body thus enveloped, forms a fervid atmosphere around him, which soon induces a copious perspiration, in the greater number of individuals. It has been observed that, in diseased parts, as for instance, in the joints of gouty people, the perspiration was longest in breaking out. When the skin is obstinate, friction and other means are used to accelerate the cutaneous discharge. When the physician judges that the perspiration has been sufficient, the patient is quickly disrobed and plunged into a cold bath, which is kept ready at the side of his bed. The first shock is very unpleasant; but that over, the invalid feels very comfortable, and when the process is likely to prove favourable, there is frequently observed on the surface of the water a kind of viscid scum, the supposed morbid matter thrown off from the body. The period of immersion in the cold bath is carefully watched, for if protracted too long it proves hurtful, or even dangerous. Some people will not bear the cold immersion above a minute—others are allowed to remain till the approach of a second shiver. Where the patient is very delicate or weak, the temperature of the bath is raised a little. In other cases, the bath is artificially depressed below the natural temperature of the water.

On emerging from the bath, the patient is quickly dressed, and immediately commences exercise, and drinks abundantly of cold water. The limit to this ingurgitation is sense of pain or weight in the stomach. The patient, although rather averse to the cold drink at first, soon becomes fond of it, and will swallow fifteen or twenty goblets with a keen relish. After the promenade and cold drink is over, a nourishing breakfast is taken. All stimulating or exciting beverages are entirely prohibited. The appetite generally becomes keen, and the digestion, even of dyspeptics, strong and effective during this course. Between breakfast and dinner is variously employed, according to the strength of the patients or the nature of the disease. Some take riding or pedestrian exercise—others gymnastics—and a few have more cold water, as a plunging or shower bath.

The dinner is to be light, and soon after mid-day. It is generally taken with a keen appetite. During the three or four hours after dinner, all exercise of mind or body is forbidden, but sleep is not to be indulged in. Towards evening, some of the stronger patients repeat the same process which they underwent in the morning; but those who are weak, or in whom the crisis is approaching, only take cold water to drink in moderation. After a slight supper the patient retires to sleep, in order that he may early resume the routine of the water-cure.

The professors of this system vary the mode of application almost infinitely—

especially the external application of the cold water, according to the general or local seat of the complaint. They act very much on the doctrine of revulsion or derivation. Thus when there are symptoms of fulness or congestion about the head or the chest, a half-bath or hip-bath of cold water is employed, disregarding the first impression of cold on the lower parts of the body, but loking to the *reaction* which is to take place there, and to the consequent derivation of blood from the head and chest. Foot-baths, cold lotions, fomentations, and poultices are variously used, according to the nature or seat of the malady.

Like the spa waters, this HYDROTHERAPEIA produces, in a great many instances, a CRISIS. For some days the patients feel themselves much more energetic and comfortable than before the course was begun; but after a time 'a veritable state of fever is produced, the result of this general effervescence.'* Then the symptoms of the complaint, whatever it may be, are all exasperated and acquire an increase of intensity—even old diseases, that were forgotten, will sometimes re-appear—but all this commotion is the precursor of a salutary crisis and a return to health. A kind of prickly heat, with itching of the skin, is a common occurrence in the course of the cure. 'The effects produced even on organic diseases by this hydro-therapeutic treatment would convince the most sceptical of its wonderful efficacy.' "—*Engel.*†

So much for Mr. Claridge being the *first person* to make known hydropathy in this country! The following passage from Dr. Johnson's book relates to the *originality* of Priessnitz' practice.

" Before proceeding farther, it will be proper to explain that the transition from a hot bath to a cold one, even in a state of perspiration, is not half so dangerous as most people imagine. It is well known that if we jump out of hot water into cold, we resist the shock, and bear the effects of the latter better than if we took the plunge without any preparation. But then there is a strong prejudice that *perspiration* is an insuperable bar to the application of cold water to the surface. If the individual has come into a state of perspiration from bodily exercise, and especially if he be fatigued or exhausted—then the cold water would be dangerous. But this is not the case, to any extent, when he is warmed either by the hot bath, or by the accumulation of heat generated in his own body. This is proved by authentic facts which have come under my own observation. Forty years ago, when the Russian troops were encamped in the islands of Guernsey and Jersey, the soldiers constructed rude stone huts or ovens along the beech, for vapour baths. Into these they put stones, and heated them by fire, when they poured water over them, and thus filled the hut with a dense vapour. When the men had continued in this rude vapour-bath till they were in a state of perspiration, they leaped into the sea, and swam about till they were tired. All this was done, partly for health, partly for pleasure. It is well-known to all northern travellers that the Russians are in the habit of steaming themselves in the vapour-baths, and then directly rolling themselves in the snow. Every one, too, must have observed postillions dashing their foaming and perspiring horses into any convenient water at the end of their journey, without the least fear of their animals being injured by the dip.

Here then is a complete counter-part, or rather prototype of the HYDRO-SUDO-PATHY, as already described. But there is one process which will appear incredible to most people—that of procuring perspiration by means of blankets wetted with cold water. Let us see whether an illustration of this may not be found. Every one who has read the Waverly Novels must have been struck with the singular practice pursued by some Highlanders (outlaws I think) who were obliged to pass many winter nights unsheltered on the freezing mountains.

---

* Dr. Engel, of Vienna † Pilgrimages to the Spas, p. 134..

When they were desirous of sleeping, they dipped their plaids in the freezing water of the nearest pool or stream, and, wrapping themselves in this dripping and gelid mantle, went quietly to sleep! So long as the plaid kept wet, the Highlander kept warm, and slept soundly; but the moment it got dry, the man was awoke by the cold, and proceeded to the brook or stream to saturate his bed-clothes again with cold water. Here we have the prototype of the German process described in the case of the girl with inflamed lungs. By what process of *reasoning* the Silesian peasant and the Celtic mountaineer, arrived at the knowledge of these curious facts, would be difficult to imagine. There was probably no reasoning in either case, but chance, observation, and experience.

It is sometimes more easy to explain a phenomenon when discovered, than to arrive at it by any process of reasoning previously. The wet plaid by confining the animal heat of the Highlander, soon occasioned a warm atmosphere around his body, which kept him comfortable. But as soon as the plaid got dry and its texture *pervious*, then the animal heat rapidly escaped, and the feeling of cold dispelled sleep." *Ib.* p. 136.

Gräeffenberg is a colony of about twenty houses, placed about half way up one of the mountains of the Sudates in Silesia, 70 miles from Breslau —260 from Berlin—200 from Dresden—and 175 from Vienna. In the valley below is the small town of Freiwaldau, where families are accommodated, and forms the fashionable wing of the hydropathic establishment. The valetudinarium itself, of Gräeffenberg, is elevated 600 feet above the town of Freiwaldau, and commands magnificent prospects; but it is badly arranged, " there being always a disagreeable smell in it—first, from the cows, which are *kept under the house;* secondly, from the public conveniences, which are on the stair-case—and thirdly, from the kitchen, which is under the saloon, up into which, the dinner is raised through a trap-door, by means of pullies" !! These three sources of malodorous, if not malarious effluvia (cows, commodes (?) and cookery) must, when united, produce a TERTIUM QUID, which the olfactories, even of a German, would not readily relish! The cows, the commodes, and the cooks indeed, ought to be subjected daily to the " water-cure," of which, we suspect, they stand in quite as much need as the valetudinarians themselves. The accommodations in the chambers of Gräeffenberg are not of the most refined kind. " A bed-stead, with straw mattress—a chest of deal drawers—a table—two chairs—a wash-hand-basin—a decanter and two glasses, comprise the whole furniture." M. Priessnitz very wisely and philosophically concludes that—" a want of comfort in the apartments is an advantage, as it induces people to be a great deal *out of doors.*" " Reading, writing, and thinking are obstacles to recovery." The expense of living at the valetudinarium is about twenty shillings a week, including the honoraria (2 florins, or 4s. to the second Hippocrates, Dr. Priessnitz) who, however, is generally presented with a florin or two extra, by the generous or the wealthy.

We shall not dilate on the biography of Priessnitz. He was the son of a farmer, and took the hint of curing his fellow-creatures by cold water, from a *Physicianer*, or, in other words, a *Cow-Doctor* in the neighbourhood. Having been run over by a cart, which fractured two of his ribs, a surgeon (?) from Freiwaldau pronounced that Hippocrates the second would never be " fit for work again." The GENIUS did not relish the prognostication, and endeavoured to cure himself.

"To effect this his first care was to replace his ribs, and this he did by leaning with his abdomen with all his might against a table or a chair, and holding his breath so as to swell out the chest. This painful operation was attended with the success he expected; the ribs being thus replaced, he applied wet cloths to the parts affected, drank plentifully of water, ate sparingly, and remained in perfect repose. In ten days he was able to go out." 58.

The fame of this "extraordinary cure" spread far and wide—"so that his house was beset with persons, rich and poor, begging his advice." We think we need not go much farther, after this specimen of the manner in which medical fame is sometimes acquired. St. John Long was an example in our own time.

Of Mr. Claridge's own liberality and good sense, the following is a specimen.

"There is no doubt that Mr. Priessnitz owes all his experience to his utter ignorance of medical science, which, indeed, is his greatest advantage; for what does the history of medicine offer, but the discouraging picture of the instability of principles, and a series of theories succeeding each other, without any one of them being able to content an upright spirit, or satisfy an inquiring mind?" 62.

Truly, "if ignorance be bliss, 'tis folly to be wise." The author before us tells us that, although Priessnitz may have some theory in his own mind, he has never disclosed it, either by oral communication, or by writing. Yet a few pages farther Mr. C. gives us a most elaborate theory of his second Hippocrates, containing no less than thirteen doctrines, or at least dogmas—of which we can only spare room for one, the XIth.

"To think of curing disease with the poison commonly called physic, must, to the reflective mind, appear paradoxical, because it is impossible to bring the physic to bear upon the dispersed and deeply-hidden diseased matter; and even if this could be done, it is quite impossible, as every chemist knows, that the morbid matter and physic should mutually dissolve each other into nothing. The consequence of such treatment with physic is, that to the old evil, a new stimulus is added, weak or strong, according to the dose and quality." 90.

Another dogma or doctrine of this second Hippocrates is, that acute diseases are, at the best, but changed into chronic, by medicines, and are then either incurable, or to be remedied only by hydropathy! Now, absurd and erroneous as these dogmas are, they will tend to confirm a considerable portion of the public in a prejudice which is daily gaining ground —namely, that the present system of polypharmacy is injurious more frequently than beneficial. It behoves the general practitioner, as we have often remarked, to change, as soon as possible, the mode of charging for medicines instead of attendance. If they do not adopt this plan, immediately that it is legalized by the pending bill, they will commit professional suicide.

The list of diseases for which the process already described is applied, contains, as usual, almost all the maladies in Cullen's or Good's Nosology. We need only specify a few; viz: gout, rheumatism, all kinds of fevers, dropsy, cancer, cholera (!!), dysentery, pneumonia (!!), scarlatina, measles, small-pox (!!), inflammation of brain, syphilis, quinzy, dyspepsia of all shapes, tic-douloureux, epilepsy, uterine hæmorrhage, &c. &c. Now, we need hardly say that, with the exception of dyspepsia, chronic rheumatism, and a few other chronic ailments, the hydropathic system will, in almost all instances, prove injurious or even dangerous. In all cases of acute inflammation—perhaps in all cases of sub-acute inflammation, the plunge from perspiration to the cold bath will be little short of insanity.

It is said that smooth water runs deep. We strongly suspect a deep current of self-interest under the spacious flow of philanthropy and humanity pervading this volume—especially through that portion of it which claims to be original. We have seen the virulent hostility which the author evinces towards the medical profession, and to physic of every kind. We have seen that a Silesian peasant, so ignorant of anatomy as not to know in which side of the body the liver is placed, has been elevated into an "astounding genius"—a "second Hippocrates," by Mr. Claridge. At the same time, it is unequivocally hinted that—"he does not see how individuals who are in *no other* way acquainted with the treatment (of hydropathy) than what they learn from books, are to carry it into execution, *without the assistance of some one who understands Mr. Priessnitz's mode of treatment*." So so! Now, if a hydropathic establishment be set up at Richmond or Norwood, where there is good air and plenty of water—and if medical knowledge be not only unnecessary, but actually prejudicial, who can more appropriately enact the part of Hippocrates the second, than Hippocrates the third—Mr. Claridge himself? Nous verrons!

Before parting, however, we may just address a few hints to any UNDERTAKER who may establish a hydropathic valetudinarium in this country. With the exception of Priessnitz, amongst his wild mountains of the Sudates, none of the various speculators, in Germany, can be said to have succeeded. And why? Not because they have lacked the "astounding genius," and the profound ignorance of the Silesian peasant, but because hydropathy could not succeed wherever there were competent judges of its merits. It was, and always will be found that in the "water-cure," that which is true is not new, and that which is new, is not only untrue but dangerous. He who practises on inflammatory complaints on hydropathic principles will find find himself, some fine morning, in Newgate, as surely as St. John Long murdered Miss Cashin. Neither will he find John Bull so tractable an animal as his cousin-*German*. John will be found to have little relish for the wet sheets and greasy blankets so much in vogue at Gräeffenberg, having quite enough of dripping garments in his own humid climate. We strongly suspect too, that Johnny will be very loth to exchange his pint of "stout" for a gallon of Thames water. Mr. Claridge, if he expects to introduce hydropathy into this country, will be found to illustrate the old adage—

"Quem Deus vult perdere prius dementat."

By outraging the feelings of a whole profession, the science and practice of which he is entirely ignorant of, he has laid the foundation for a vigilance over his proceedings, which will effectually check all his prospects—if indeed he be insane enough to attempt the personification of Priessnitz in a country like this.

